# Post-transcriptional and post-translational regulations of drought and heat response in plants: a spider’s web of mechanisms

**DOI:** 10.3389/fpls.2015.00057

**Published:** 2015-02-11

**Authors:** Davide Guerra, Cristina Crosatti, Hamid H. Khoshro, Anna M. Mastrangelo, Erica Mica, Elisabetta Mazzucotelli

**Affiliations:** ^1^Genomics Research Centre, Consiglio per la Ricerca e la Sperimentazione in Agricoltura, Fiorenzuola d’Arda, Piacenza, Italy; ^2^Department of Agronomy and Plant Breeding, Ilam University, Ilam, Iran; ^3^Cereal Research Centre, Consiglio per la Ricerca e la Sperimentazione in Agricoltura, Foggia, Italy

**Keywords:** stress tolerance, drought stress, heat stress, alternative splicing, miRNA-mediated gene silencing, ubiquitination, sumoylation

## Abstract

Drought and heat tolerance are complex quantitative traits. Moreover, the adaptive significance of some stress-related traits is more related to plant survival than to agronomic performance. A web of regulatory mechanisms fine-tunes the expression of stress-related traits and integrates both environmental and developmental signals. Both post-transcriptional and post-translational modifications contribute substantially to this network with a pivotal regulatory function of the transcriptional changes related to cellular and plant stress response. Alternative splicing and RNA-mediated silencing control the amount of specific transcripts, while ubiquitin and SUMO modify activity, sub-cellular localization and half-life of proteins. Interactions across these modification mechanisms ensure temporally and spatially appropriate patterns of downstream-gene expression. For key molecular components of these regulatory mechanisms, natural genetic diversity exists among genotypes with different behavior in terms of stress tolerance, with effects upon the expression of adaptive morphological and/or physiological target traits.

## PLANT RESPONSES TO DROUGHT AND HEAT CHALLENGES

Drought and heat stress widely threaten plant growth, development and crop yield. Plants have evolved morphological and physiological adaptations, and molecular responses activated upon stress perception to cope with environmental constraints. These mechanisms contribute to (1) avoid cellular dehydration and overheating by maintaining a high water potential in the tissues and a permissive temperature during drought and heat stress respectively, or (2) to survive water deficiency and high temperature by activating tolerance strategies.

Drought and heat tolerance are complex quantitative traits involving many different physiological parameters associated with plant adaptability to unfavorable environments (Cattivelli et al., 2008; Cossani and Reynolds, 2012). Examples are the improvement of water balance through stomatal closure, deep roots, cuticular resistance and reduced leaf area, or the counteraction to hazardous effects of stress by osmotic adjustment, antioxidant defense, and membrane thermostability, in addition to phenological changes like high initial growth rate for a rapid ground cover, early maturity and late senescence. Some traits lead to a reduced total seasonal evapotranspiration, but these are often associated with reduction of yield potential (Cattivelli et al., 2008). Thus, the adaptive significance of some stress-related traits seems more related to survival than to agronomic improvement. Moreover, even if plant responses to drought and heat show common features, some traits for heat avoidance may favor dehydration. Leaves evaporate water to keep physiological temperatures, but a prolonged opening of stomata could be detrimental to water potential. This complexity of interlinked responses, in addition to the gene-environment interactions, has always prevented classical breeding from quickly advancing in the development of heat and, above all, drought resistant genotypes.

Physiological and morphological adaptations to environmental constraints are associated with wide transcriptional changes controlled by sophisticated molecular mechanisms. The powerful influence of these regulatory mechanisms is just emerging together with their role of fine-tuning the expression of stress-related phenotypic traits, thus striking a balance between contrasting responses. In the past three decades, important advances have been achieved in understanding the molecular basis underpinning stress tolerance, through both the identification of differentially expressed genes and cloning of hundreds of stress-related mutants. However, the identification of the genetic basis of stress-related phenotypic traits lags behind, since relatively few genes/QTLs controlling morphological and/or physiological adaptive traits have been cloned (Masle et al., 2005; Salvi et al., 2007; Chen et al., 2011; Uga et al., 2013).

Recent research has highlighted further levels of molecular regulation of gene expression based on post-transcriptional and post-translational processes. The mRNA population can be affected both quantitatively and qualitatively at various steps during RNA processing. Alternative splicing (AS) has important consequences for the availability of different kinds of transcripts, and ultimately of proteins (Mastrangelo et al., 2012). RNA silencing is an additional mechanism to down-regulate the amount of specific transcripts through the action of different classes of small RNAs (Kruszka et al., 2012). RNA regulation particles, namely processing bodies and stress granules, are emerging as types of RNA regulation involved in mRNA degradation and mRNA stabilization, respectively (Bailey-Serres et al., 2009). Control of translational initiation may widely repress general gene expression, with the exception of specific subsets of mRNAs which escape such repression and remain actively translated (Muñoz and Castellano, 2012). After translation, a plethora of molecules can bind proteins and modify their activity, sub-cellular localization and half-life. Protein phosphorylation has been recognized for decades as one of the major mechanisms for the transmission of stress signals, while other types of modifications, like S-nitrosylation, are just emerging (Romero-Puertas et al., 2013). Ubiquitin and SUMO conjugations are arising as major post-translational regulatory processes in all eukaryotes (Vierstra, 2012). These modifications may act on the same transcript/protein target at different levels during the transcriptional/translational process. Thus, the combination of all of these modifications determines the final amount of the target and the effect on the corresponding process/phenotypic trait.

This review focuses on four regulatory mechanisms: AS, miRNA-mediated gene silencing, ubiquitination and sumoylation. Indeed, the research in recent years has led to breakthroughs on these topics, making them to stand out over other processes as important fine-tuning regulatory mechanisms in the plant response to stress. Many transcript/protein targets of these post-transcriptional and post-translational modifications have been identified and they are often regulators of cellular processes, including signaling components and transcription factors (TFs). Moreover, functional studies have demonstrated their effective role in drought and heat stress tolerance. The emerging picture identifies these post-transcriptional and post-translational mechanisms and their interactions as pivotal regulatory systems of the transcriptional changes related to cellular and plant stress response. This regulatory web ensures spatially and temporally appropriate patterns of downstream gene expression and ultimately the fine-tuning of the expression of physiological or morphological target traits. Finally, preliminary evidence is emerging about natural genetic diversity of key molecular components of these regulatory mechanisms, further adding new operational layers for tuning target traits. Thus, these post-transcriptional and post-translational mechanisms represent new potential targets for the development of novel genotypes with enhanced stress tolerance. They could lay the foundations of a new generation of transgenics or support the molecular breeding of new genotypes carrying the best combinations of the corresponding alleles.

## ALTERNATIVE SPLICING TARGETS BOTH REGULATORY COMPONENTS AND DOWNSTREAM STRESS-RELATED GENES OF THE PLANT RESPONSE TO DROUGHT AND HEAT

**FIGURE 1:**
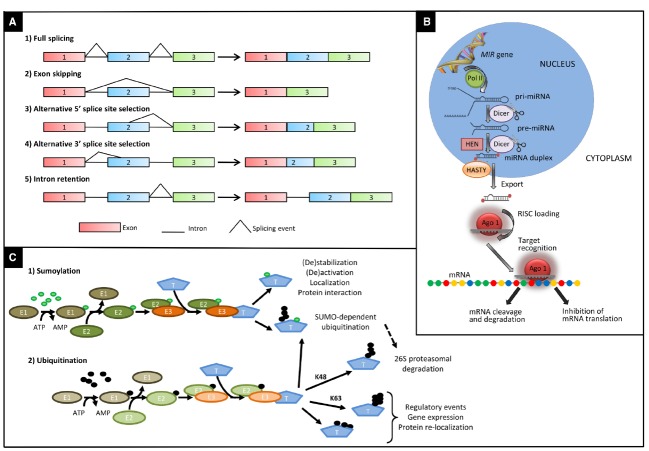
**Main types of post-transcriptional and post-translational modifications affecting the molecular response to drought and heat.** **(A)** Main types of alternative splicing events: 1, fully spliced transcript; 2, exon skipping; 3, alternative 5′ splice site; 4, alternative 3′ splice site; 5, intron retention. **(B)** Major steps in the biogenesis of miRNAs in plants. Primary transcripts (pri-miRNA) of plant-miRNAs are transcribed by RNA polymerase II (POL II) from their own chromosomal loci. Pri-miRNAs fold in a stem-loop (hairpin) secondary structure. Following transcription, pri-miRNAs are converted to miRNA-precursors (pre-miRNA) and subsequently to a miRNA/miRNA* duplex by RNAse III enzyme Dicer like1 (DCL1). Then the miRNA/miRNA* duplex is methylated at the 3′-ends by the methyl transferase HEN1 and exported to the cytoplasm via exportin-5-like protein Hasty. One strand of the methylated miRNA/miRNA* duplex is selectively incorporated in the RNA-induced silencing complex (RISC) containing Argonaute (AGO) to repress or cleavage target mRNA in sequence specific manner. **(C)** Enzymatic steps of sumoylation (1) and ubiquitination (2) and main fates of ubiquitinated proteins. The enzymatic cascade, composed of an ubiquitin or SUMO activating enzyme (E1), a conjugating enzyme (E2), and a ligase enzyme (E3), mediates the labeling of a specific target protein (T) by SUMO (green circles) or ubiquitin (black circles). In the latter case, proteins can be marked with a K48-linked ubiquitin chain of at least four ubiquitin molecules to be degraded via the 26S proteasome. Alternatively, they can be ubiquitinated via K63 residues of ubiquitin or multi-monomeric ubiquitination leading to regulatory events. In addition to a wide range of other effects, sumoylation can also act as a signal for a subsequent ubiquitination, by tagging target protein for proteasome-mediated degradation.

Alternative splicing is a process in which two or more different transcripts are generated from the same pre-mRNA molecule by using different splice sites. Four main types of AS are known: exon skipping, alternative 5’ and alternative 3’ splice sites, and intron retention (Figure 1; Reddy, 2007). The ratio of intron retention to exon skipping varies substantially among the kingdoms, with higher values in plants compared to multicellular animals (McGuire et al., 2008). The retention of one or more introns can lead to the insertion of an in-frame premature termination codon within the transcript, making it a target for degradation by nonsense-mediated decay (NMD), an mRNA surveillance mechanism believed to prevent accumulation of truncated, and potentially harmful, proteins (Soergel et al., 2006). AS coupled with NMD represents a mechanism for fine-tuning the amount of functional transcripts in a cell. Otherwise, mRNAs can be translated into truncated proteins, which can contribute to control the amount of the functional protein that is produced, or might themselves have functional roles, as demonstrated for many resistance genes in plants (Dinesh-Kumar and Baker, 2000; Zhang and Gassmann, 2003; Staal and Dixelius, 2008).

From 20 to 70% of expressed genes have been shown to undergo AS depending on the species considered (Mastrangelo et al., 2012; Reddy et al., 2013; Staiger and Brown, 2013). Genome-wide analyses of AS have found that stress-related gene ontology categories are over-represented in the genes subjected to AS, while housekeeping cellular functions are mainly controlled at transcriptional level (Ner-Gaon et al., 2004; Eichner et al., 2011). Moreover, many genes undergoing AS are involved in the regulation of the plant response to abiotic stress (Filichkin et al., 2010).

Many studies have been focused on AS events with a functional role in the stress response. These may fine-tune the expression of regulatory and structural genes by producing non-functional transcripts in control conditions, which are replaced by active variants upon stress, or *vice versa* switching off the production of a functional form upon stress. The splicing of transcripts encoding the signaling protein DROUGHT RESPONSIVE ANKYRIN1 (DRA1) in *Arabidopsis* is an example of intron retention involved in drought tolerance. *DRA1* encodes a transmembrane protein with an ankyrin-repeat motif whose disruption leads to increased drought-stress tolerance. At least two splicing variants of *DRA1* transcripts were identified. In response to drought stress, the levels of *DRA1* transcripts retaining the second and the third introns increase, whereas these introns are removed under control conditions (Sakamoto et al., 2013).

**FIGURE 2:**
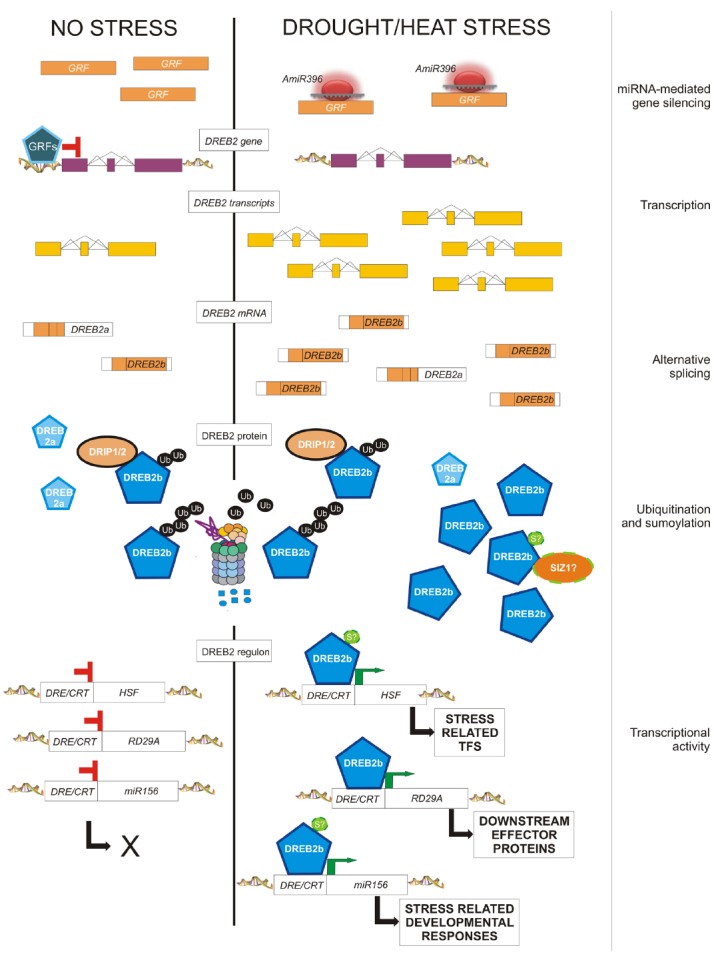
**Post-transcriptional and post-translational modifications eventually affecting the amount and activity of DREB2 in response to drought and heat.** In the absence of stress, cells produce a small amount of a short *DREB2* mRNA encoding truncated non-functional proteins (DREB2a), and full mRNA transcripts (*DREB2b*) encoding functional proteins (DREB2b). Moreover, GRFs repress the expression of the *DREB2* gene. Upon stress perception, *miR396* down-regulates *GRF* transcripts, thus allowing the induction of the transcription of the *DREB2* gene, as well as exon skipping of corresponding transcripts, leading to full *DREB2b* mRNAs and functional proteins. The constitutively expressed *DRIP1* encodes an ubiquitin ligase that can always target the functional DREB2b protein for 26S proteasome-mediated degradation, likely through ubiquitination in its negative regulatory domain. This domain is predicted to also contain a SUMO consensus sequence. Thus, sumoylation could contribute to DREB2 activation/ protection from degradation. Active DREB2 proteins are responsible for the stress-induced expression of (a) TFs (like HSFs), in turn responsible for secondary stress-related changes of gene expression, (b) downstream genes, which account for protective or repairing functions, and (c) stress-related miRNAs, responsible for developmental changes in response to environmental conditions.

The expression of *DEHYDRATION-RESPONSIVE ELEMENT BINDING 2B* (*DREB2B*) is regulated by AS in response to stress in rice, wheat, barley and maize (Xue and Loveridge, 2004; Egawa et al., 2006; Qin et al., 2007; Matsukura et al., 2010). *OsDREB2B* belongs to the well-characterized *CBF/DREB* family of TFs, which control tolerance to dehydration and low temperature in many plant species (Saibo et al., 2009; Chen et al., 2010; Morran et al., 2011). In rice, in the absence of stress, cells produce the non-functional transcript of *OsDREB2B*, which is rapidly converted to the full-length, fully functional transcript by changing the splicing pattern of the gene (Figure 2). This regulation saves the time necessary for transcriptional activation and pre-mRNA accumulation and avoids the negative effects and the metabolic costs of the constitutive expression on plant growth.

Alternative splicing can also act as a mechanism for auto-regulation of TFs in response to abiotic stresses. A heat-induced truncated form of the HEAT-SHOCK TRANSCRIPTION FACTOR A2 (HsfA2), a key regulator in response to heat stress in *Arabidopsis*, is produced by AS. This truncated form (*S-HsfA2*), but not the full-length form, binds to the *HsfA2* promoter to activate its own transcription in a positive auto-regulatory loop (Liu et al., 2013). Severe heat shock-induced AS events also occur in four other *Arabidopsis HSF* genes, and this suggests that it is a common feature for AtHsfs.

A lot of evidence indicates a role of AS in the stress-related modulation of the components of the splicing machinery. For instance, nearly 100 different transcripts are produced from 16 genes encoding serine/arginine (SR) splicing factors in *Arabidopsis* by AS following developmental and environmental stimuli (Isshiki et al., 2006; Palusa et al., 2007). SR proteins are a highly conserved family of RNA-binding proteins that both execute and regulate pre-mRNA splicing in different plant tissues, at different developmental stages, and in response to abiotic stresses (Duque, 2011). Intriguingly, SR proteins can promote AS of their own transcripts, as well as of other gene products.

Alternative splicing has also been shown to control the expression of genes belonging to the circadian clock. This mechanism synchronizes some physiological processes with predictable environmental changes. Light and temperature are the main environmental factors that regulate the circadian clock, but recent studies have demonstrated a clear regulation of clock genes in response to drought (Schöning et al., 2008; Filichkin et al., 2010). The *CIRCADIAN CLOCK ASSOCIATED 1* (*CCA1*) gene was shown to be regulated by drought in soybean and *Arabidopsis* (Huang et al., 2008; Marcolino-Gomes et al., 2014). Moreover, alternatively spliced forms are differentially accumulated in response to drought, cold and light (Filichkin et al., 2010). The *GLYCINE RICH PROTEIN* (*GRP*) *7* and *GRP8* are other *Arabidopsis* clock genes acting as slave oscillators downstream of the circadian clock through feedback loops in which alternatively spliced transcripts regulate their own expression. Furthermore, alternatively spliced forms of *AtGPR7* regulate AtGRP8 accumulation and *vice versa* in response to low temperature (Schöning et al., 2008). Transgenic rice plants over-expressing *AtGRP7* and *AtGRP2* have a higher grain number per panicle, and consequently a higher grain yield under drought stress conditions (Yang et al., 2014). So far, it is still not clear if AS is involved in the regulation of these clock genes in response to drought, as demonstrated for *CCA1*.

Alternative splicing-variants have also been described for many other regulatory genes of the drought response, but these variants still wait to be functionally characterized. For instance, AS has been reported for the TF MYB60, which is involved in stomatal regulation and control of root growth under drought in *Arabidopsis* (Oh et al., 2011), and for the protein kinase OPEN STOMATA 1 (OST1), involved in the abscisic acid (ABA) mediated response to drought in *Brassica oleracea* (Wang et al., 2013).

Finally, downstream stress-related genes can also be regulated by AS in response to drought. Two alternative isoforms were studied for the *Arabidopsis* gene *ZINC-INDUCED FACILITATOR-LIKE 1* (*ZIFL1*), coding for a transporter involved in the polar auxin transport (Remy et al., 2013). The full-length ZIFL1.1 protein and a truncated splice isoform, ZIFL1.3, localize to the tonoplast of root cells and to the plasma membrane of leaf stomatal guard cells, respectively. The ZIFL1.3 isoform mediates drought tolerance by regulating stomatal closure. Thus, determining the sub-cellular and tissue distribution of two isoforms, AS dictates a dual function for the ZIFL1 transporter.

## miRNAs CONNECT REGULATORY PATHWAYS FOR THE FINE-TUNING OF ABIOTIC STRESS RESPONSE AND PLANT GROWTH

The burst of deep sequencing technologies in the last few years has allowed the identification and quantification of many classes of small RNAs involved in gene regulation of different biological processes (e.g., plant development, cell proliferation, biotic, and abiotic stress responses), revealing an additional level of post-transcriptional control of gene expression. Much relevant evidence of small RNA-based regulatory mechanisms in drought and heat response refers almost entirely to miRNAs. They constitute a class of endogenous small non-coding RNAs (20–24 nt long) that act by sequence pairing to the mRNAs of their target genes and inhibiting their translation or cleaving them (Axtell, 2013; Figure 1). A comprehensive repository for miRNA regulatory mechanisms involved in plant response to abiotic stresses is offered by the PASmiR database (Zhang et al., 2013b).

Several functional studies addressed the role of miRNAs in the plant tolerance to drought and heat, showing a strong impact on different stress-related traits, from growth control to stomatal closure, root development, osmo-protection and antioxidant defense, as well as on the crosstalk between different hormonal pathways (e.g., auxin and ABA signaling). *miR398* is an example of miRNA associated with antioxidant defense. This miRNA regulates three Cu/Zn *SUPEROXIDE DISMUTASE* (*CSD*) genes. CSD enzymes scavenge reactive oxygen species (ROS) upon oxidative stress. *miR398* may have different roles depending on the abiotic stress. Upon heat exposure, *miR398* is induced by HSFs, thus repressing the expression of its target genes, *csd1, csd2*, and *ccs* (a gene encoding a copper chaperone for both CSD1 and CSD2). Transgenic plants carrying *miR398*-resistant forms of *CSD1*, *CSD2*, or *CCS* are more sensitive to heat stress being defective in heat-responsive gene regulation (Guan et al., 2013). On the other hand, *miR398* is down-regulated upon water stress or high salinity in rice, releasing the negative regulation of ROS scavenging enzymes (Lu et al., 2010). Similar evidence has been reported in *Medicago truncatula* and wild emmer (Trindade et al., 2010; Kantar et al., 2011).

More often stress-related miRNAs are involved in regulatory networks connecting the progression of plant growth with abiotic stress responses. For instance, many genes in the auxin signaling cascade have been identified as targets of drought and ABA responsive miRNAs. For example, an increased level of *miR393* in *Arabidopsis* under drought results in the down-regulation of the auxin receptor *TRANSPORT INHIBITOR RESPONSE 1* (*TIR1*), which, in turn, may reduce plant growth, a general response to stress conditions (Chen et al., 2012). This interaction is conserved across plant species, with interesting effects. Indeed, *miR393* over-expressing rice plants showed increased tillers and early flowering, two traits that are often associated with yield, although without any improvement in drought tolerance (Xia et al., 2012). Another example involves *miR160* and *miR167*. Their up-regulation when plants are exposed to stress reduces the transcript levels of *AUXIN RESPONSE FACTORs* (*ARFs*), which in turn cause the attenuation of plant growth and development. Under normal conditions, basal levels of these miRNAs are sufficient for fine-tuning *ARF* levels, allowing the transcription of auxin responsive genes and plant growth (Sunkar et al., 2012; Kumar, 2014). It has been demonstrated that *miR160* and *miR167* are also responsible for the subtle balance of those *ARF* transcripts controlling the formation of adventitious roots (Gutierrez et al., 2009). *miR160* targets the negative regulator *ARF17*, while *miR167* targets the positive regulators *ARF6* and *ARF8.* The regulatory network is made more complex since the three *ARF*s regulate each other’s expression at both transcriptional and post-transcriptional levels by modulating *miR160* and *miR167* availability.

*GROWTH REGULATING FACTORS* (*GRFs*) are targeted by *miR396* in different plant species, modulating organ growth in normal and stress conditions. In* Arabidopsis*, GRF7 binds to the *DREB2A* promoter, repressing its expression and acting as a negative regulator of osmotic stress responsive genes (Kim et al., 2012, Figure 2). Upon stress, *GRFs* are down regulated by *miR396*, reducing plant growth and allowing stress tolerance mechanisms (Liu et al., 2009). Both transgenic plants over-expressing *miR396* or *amiG7* (artificial miRNA against GRF7) show enhanced dehydration tolerance driven by osmotic-stress inducible genes, growth reduction and morphological modifications (Liu et al., 2009; Kim et al., 2012).

Other miRNAs are responsible for the regulation of stress-related TFs in the absence of stress, with this regulation being released through a decrease of miRNA expression upon stress perception. For instance, the *Arabidopsis miR169a* is involved in stomatal control through its action on the transcripts of the A subunit of the *NUCLEAR FACTOR Y* (*NFYA5*; Li et al., 2008). NFYA5 is a positive regulator of drought tolerance that is expressed in guard cells, where it controls stomatal aperture. It also regulates a number of drought stress-responsive genes, such as glutathione S-transferase and peroxidase. The down-regulation of *miR169a* upon drought or ABA treatments contributes to the strong induction of *NFYA5*. Analogously, *miR167a* targets *IAA-ALA RESISTANT 3* (*IAR3*), an essential gene for the release of bioactive auxin (IAA) which is the central phytohormone of root development (Kinoshita et al., 2012). High osmotic stress causes a decrease in *miR167a* levels, and the subsequent accumulation of *IAR3* mRNAs, thus allowing stress-induced root architecture changes. Indeed, transgenic plants expressing a cleavage-resistant form of *IAR3* mRNA showed increased lateral root development.

The heat stress response, as well, results in down-regulation of several miRNAs. *miR159* expression, as an example, is rapidly reduced in bread wheat exposed to heat stress (Wang et al., 2012). *GAMYB* TFs, the main targets of *miR159*, have potential roles in heat tolerance. Indeed, transgenic wheat plants over-expressing *miR159* or *Arabidopsis* double mutant *myb33 myb65* are more susceptible to high temperatures.

Otherwise, stress-related TFs can affect the expression of small RNAs in multiple ways, these small RNAs are then responsible for the positive regulatory effect of the TFs themselves. For instance, deep sequencing of small RNAs in barley over-expressing *TaDREB3* identified a number of transgenic specific miRNAs that are likely activated by *TaDREB3*, as well as miRNAs absent in transgenic barley and probably suppressed by the TF (Hackenberg et al., 2012). Moreover, these differentially expressed miRNAs are connected to each other in complex networks. Such a modulation of small RNAs could be coupled with the function of *TaDREB3* to contribute to the increased drought tolerance of transgenic barley plants over-expressing *TaDREB3. miR156* and *miR172* are among the miRNAs modulated in *TaDREB3* over-expressing barley, respectively up- and down-regulated (Hackenberg et al., 2012). *miR156*, one of the most highly conserved plant miRNAs, is essential for the juvenile phase and acts by repressing the expression of distinct *SQUAMOSA PROMOTER BINDING PROTEINS LIKE* (*SPL*) which control flowering time, phase change and leaf initiation rate. *miR172*, which controls adult epidermal identity through target *APETALA2* (*AP2*)-*LIKE* genes, shows a pattern of expression opposite of *miR156*, with low expression levels during the juvenile phase and high expression during the adult phase. A regulatory network proposed by Wu et al. (2009) shows the regulation of *miR172* expression through SPL9, target of *miR156*, a mechanism responsible for early flowering phenotypes in drought conditions.

## UBIQUITINATION REGULATES MULTIPLE ASPECTS OF DROUGHT AND HEAT STRESS RESPONSE

Ubiquitination is the mechanism through which the small ubiquitin molecule is conjugated to a protein substrate. The labeling of a protein with ubiquitin is accomplished by the action of three enzymes, the ubiquitin activating enzyme (E1), the ubiquitin conjugating enzyme (E2), and the ubiquitin ligase (E3) (Vierstra, 2009, Figure 1). E3s are responsible for recruiting target proteins and, therefore, for conferring specificity to the ubiquitination process. Ubiquitinated proteins have several different fates, the most common one being degradation by the 26S proteasome, but changes in their sub-cellular localization or activity are also potential fates (Hershko and Ciechanover, 1998).

Analyses of stress-related mutants have revealed many ubiquitin ligases accounting for regulatory roles in plant tolerance to abiotic stress, especially drought, both in *Arabidopsis* and crop species (Cho et al., 2008; Lyzenga and Stone, 2012; Stone, 2014). Some of their targets have been identified, allowing specific placement of E3 ligases in stress response pathways. A small regulatory protein network supporting drought tolerance has been discovered for the durum wheat E3 ubiquitin ligase RING FINGER 1 (TdRF1; Guerra et al., 2012). The best described E3 ligases modulate the abundance of TFs, thus affecting stress-related changes in gene expression. E3 ligases may prevent transcription activity by targeting TFs and inducing their degradation in the absence of stress. This is the case of the *Arabidopsis* E3 ligases DREB INTERACTING PROTEIN 1 (DRIP1) and DRIP2, which mediate the degradation of the drought-induced DREB2A (Qin et al., 2008; Figure 2). Alternatively, ubiquitin-dependent degradation can also lead to an attenuation of stress signaling. This is the case of the E3 ligases RING DOMAIN LIGASE (RGLG) 1 and RGLG2, which regulate the abundance of the drought-induced ETHYLENE RESPONSE FACTOR 53 (Cheng et al., 2012). Other E3s target downstream genes, for instance the *Arabidopsis* drought-induced RING MEMBRANE-ANCHOR 1 (Rma1H1) that contributes to drought tolerance by regulating the levels of aquaporins (Lee et al., 2009). The involvement of non-proteolytic function of ubiquitin modification has also been reported; in particular, mono-ubiquitination and lysine-63 linked poly-ubiquitination. For instance, the rice HEAT AND COLD INDUCED 1 (OsHCI1), the only known E3 ligase involved in heat response, monoubiquitinates some nuclear proteins upon exposure to heat treatment (Lim et al., 2013). Subsequently, these proteins translocate from the nucleus to the cytoplasm, probably promoting heat stress tolerance.

An increasing number of studies indicate a strong link between signaling of ABA, ubiquitination and drought tolerance, at many levels of the signaling cascade. Ubiquitination affects ABA signaling beginning with the early stages of biosynthesis, acting either as negative or positive regulator. On one hand, in *Arabidopsis* the E3 ubiquitin ligase DROUGHT TOLERANCE REPRESSOR (DOR) negatively regulates ABA biosynthesis in guard cells. Indeed, *dor* mutants show increased cellular ABA levels due to the enhanced expression of *9-CIS-EPOXYCAROTENOID DIOXIGENASE 3* (*NCED3*), and an exacerbated stomatal closure. As a result, *dor* plants are more tolerant to drought stress (Zhang et al., 2008). ABA biosynthesis is negatively regulated in a similar way by the SENESCENCE-ASSOCIATED UBIQUITIN LIGASE 1 (SAUL1) that targets the enzyme converting abscisic aldehyde to ABA, the ARABIDOPSIS ALDEHYDE OXIDASE 3 (AAO3), for proteasome-dependent degradation (Raab et al., 2009). On the other hand, a positive role of ubiquitination on ABA biosynthesis has been demonstrated for the E3 ubiquitin ligase XERICO, whose increased expression determines the induction of *NCED3*, leading to increased drought tolerance (Ko et al., 2006).

A further level of regulation exerted by ubiquitination on ABA signaling occurs during ABA perception. In *Arabidopsis*, three ABA receptors, PYRABACTIN RESISTANCE LIKE (PYLs) proteins, are targeted for proteasomal degradation by the E3 ubiquitin ligase DET1-DDB1-ASSOCIATED1 (DDA1; Irigoyen et al., 2014). Intriguingly, DDA1-mediated destabilization of PYLs is counteracted by ABA, which protects PYLs from ubiquitination. Such a mechanism may therefore serve to minimize the adverse effects of continued ABA responses (such as reduced growth and stomatal closure) under non-stress conditions, and sustain ABA signaling when needed.

Downstream of ABA signaling, ubiquitination targets several TFs regulated by ABA. For instance, the E3 ubiquitin ligase KEEP ON GOING (KEG) interacts with and targets ABRE BINDING FACTOR (ABF) 1 and ABF3 for proteasome mediated degradation, and such degradation is delayed in response to ABA (Chen et al., 2013).

Besides ABA signaling, ubiquitination controls other traits affecting drought and/or heat tolerance. For instance, some ubiquitin ligases act on leaf senescence. On one hand, in *Arabidopsis*, the ubiquitin ligase UPL5 counters senescence through the ubiquitination and degradation of WRKY53, a positive regulator of senescence (Miao and Zentgraf, 2010). On the other hand, the ORE9 ubiquitin ligase promotes leaf senescence through a still undiscovered mechanism (Woo et al., 2001). Other ubiquitin ligases regulate the plant water status by controlling some morphological structures. The *cer9* mutant in the E3 ubiquitin ligase ECERIFERUM9 (CER9) better tolerates drought through an increased accumulation of cutin, which in turn reduces water transpiration (Lü et al., 2012; Zhao et al., 2014). On the contrary, the E3 ligase XB3 ORTHOLOG 2 IN ARABIDOPSIS THALIANA 32 (XBAT32) may potentially improve the plant water status by acting as a positive regulator of lateral root development (Prasad et al., 2010). This effect is associated with the XBAT32 mediated degradation of the enzymes AMINOCYCLOPROPANE-1-CARBOXYLIC ACID SYNTHASES (ACS) 4 and ACS7, which catalyze the rate limiting step of ethylene biosynthesis, which in turn inhibits lateral root production. Auxin, instead, is a key promoter of lateral root growth and the adjustments of its distribution in root meristems are important for environmentally controlled adaptation of root growth. The PIN-FORMED (PIN) proteins are carriers instrumental for directional cellular efflux of auxin. It has been demonstrated that ubiquitination controls both the proteolytic turnover of PIN2 by Lys48-linked polyubiquitination, and its endocytosis and vacuolar targeting by Lys63-linked polyubiquitination (Leitner et al., 2012).

## GLOBAL AND SPECIFIC ROLES OF SUMOYLATION IN THE PLANT RESPONSE TO DROUGHT AND HEAT

Sumoylation is a reversible post-translational modification of protein substrates based on the covalent conjugation of the SUMO (Small Ubiquitin-like MOdifie) peptide. Sumoylation can mask and/or add interaction surfaces, or induce conformational changes, resulting in sub-cellular re-localization, changes in enzymatic activity or protection from ubiquitin-mediated degradation. Similarly to ubiquitination, catalysis of SUMO conjugation involves activating enzymes (E1), conjugating enzymes (E2), and E3 ligases; moreover, SUMO specific proteases de-conjugate the substrates making sumoylation a transient modification (Figure 1). A few E3 ligases accomplish all modifications. Indeed, just two E3s, SAP and Miz 1 (SIZ1) and Methyl Methanesulfonate-Sensitivity protein 21 (MMS21)/HIGH PLOIDY 2 (HPY2), have been identified in *Arabidopsis* (Miura et al., 2005; Huang et al., 2009; Ishida et al., 2009). Sumoylation often occurs on the lysine of a specific consensus sequence, however, other modifications, such as phosphorylation, may regulate the sumoylation of a substrate (Park and Yun, 2013). Instead, SUMO peptidases are responsible of SUMO deconjugation and therefore determine the dynamic aspects of this modification. SIZ1 is the main regulator of accumulation of the SUMO conjugates in response to stress (Yoo et al., 2006; Catala et al., 2007; Park et al., 2010), while MMS21 is partially responsible for conjugation upon ABA treatment (Zhang et al., 2013a).

The phenotypic analyses of *siz1* e *mms21* mutants indicate a critical role of sumoylation in plant tolerance to drought and heat, in addition to other environmental stresses. *Siz1* mutants lack basal thermotolerance (Yoo et al., 2006), show enhanced drought tolerance associated with reduced stomatal aperture through a salicylic acid-induced accumulation of ROS (Miura et al., 2012), and also show alteration in the expression of a wide variety of drought responsive genes (Catala et al., 2007). *mms21* mutants display improved drought tolerance associated with increased sensitivity to ABA (Zhang et al., 2013a). Indeed, MMS21 acts as a negative regulator of ABA and drought-induced stress responsive genes. Another line of evidence of the role of sumoylation in plant response to abiotic stress is the significant early accumulation of SUMO conjugated proteins triggered by: dehydration, in an ABA-independent manner (Catala et al., 2007); ABA treatments (Chaikam and Karlson, 2010; Zhang et al., 2013a); mild heat stress; and other environmental stresses as well (Kurepa et al., 2003). Moreover, it has been shown that increased sumoylation levels attenuate the ABA mediated growth inhibition and amplify the induction of ABA- and stress-responsive genes such as *RD29A* (Lois et al., 2003).

Recently, three different research strategies based on proteomic and interactomic analyses have uncovered a deep catalog of 350 SUMO targets and SUMO-interacting proteins in *Arabidopsis* (Budhiraja et al., 2009; Elrouby and Coupland, 2010; Miller et al., 2010), including those accumulated after exposure of plants to heat and oxidative stresses (Miller et al., 2013). Most of the SUMO substrates are known or predicted to regulate nuclear activities, including RNA processing, DNA methylation, chromatin remodeling, DNA repair, and gene transcription. This last nuclear activity refers to both co-repressors (i.e., LEUNIG/TOPLESS) and TFs required for developmental and/or stress-induced gene expression. Thus, these SUMO targets suggest that sumoylation is a regulatory mechanism of transcriptome changes and is an epigenetic marker that globally affects the gene expression and chromatin stability associated with stress responses. Sumoylation could act as a general and conserved stress response affecting basal cellular functions. SUMO conjugation to TFs and to LEUNIG/TOPLESS co-repressors, regulating plant development, may suggest a role for sumoylation in the decreased rate of plant growth and/or developmental transitions generally observed upon stress treatments. Components of DNA repair machinery can, in turn, facilitate plant recovery from damage elicited by the oxidative component of many environmental stresses.

Some stress-related TFs have been also identified as SUMO conjugates. Evidence for sumoylation of HSFs at high temperatures come from both plants, mainly the regulator of acquired thermotolerance AtHsfA2 (Cohen-Peer et al., 2010) in addition to others (Miller et al., 2010, 2013), and from animals (HSF1, Hong et al., 2001; HSF2, Anckar et al., 2006), with both negative and positive consequences for their transcriptional activity. So far, no drought-related TFs are known to be sumoylated. However, SUMO E3 ligase SIZ1 negatively controls ABA signaling through the sumoylation of ABA-INSENSITIVE 5 (ABI5; Miura et al., 2009) and of MYB30 (Zheng et al., 2012). Moreover, searching for the SUMO consensus sequence through *in silico* analysis (SUMO plot, http://www.abgent.com/sumoplot) predicted a highly scored sumoylation site in the negative regulatory domain of the DREB2 protein, which could account for protection from ubiquitination (Figure 2).

## NETWORKS OF POST-TRANSCRIPTIONAL AND POST-TRANSLATIONAL REGULATIONS

Evidence indicates that there are different kinds of interactions between post-transcriptional, post-translational regulatory networks, and stress-related transcriptional changes. As illustrated in Figure 2, these modifications may all target the same transcript/protein at different levels. Therefore, the final outcome, that is the amount/activity of a downstream stress-related transcript/protein, is the result of an upstream cascade of several regulatory events. The transcriptional activation of stress-related genes responds to a signal of incipient main stress. Subsequently, post-transcriptional and post-translational modifications may influence the corresponding transcripts/proteins in order to integrate other signals, including specific features of the main stress (i.e., duration), and/or signal of secondary stresses or environmental conditions (i.e., photoperiod), and/or internal growth/developmental signals (i.e., hormonal pathways). Notably, both AS, through intron retention, and miRNAs and ubiquitin ligases, as well, negatively impact their targets, and, therefore, may attenuate the first gene activation triggered upon stress perception.

In addition to interacting at target levels, post-transcriptional and post-translational regulatory networks may influence each other. Recent studies revealed a tight interaction between AS events and miRNAs; different forms of a given miRNA can be produced from the same gene as a consequence of AS, or AS can modify the binding site of the miRNA in the target transcript. In *Arabidopsis*, the case of heat-induced pre-mRNA splicing participating in miRNA processing of the intronic *miR400* may illustrate the first type of interaction (Yan et al., 2012). *miR400* is co-transcripted with its host gene *At1g32583*. Under stress conditions, an AS event occurs in the intron carrying the *miR400* resulting in a greater accumulation of *miR400* primary transcripts at the expense of the level of mature *miR400*. Intriguingly, *Arabidopsis* plants over-expressing *miR400* are more sensitive to heat stress. Thus, the heat-induced AS event acts as a negative regulatory mechanism of the *miR400* expression with a positive effect on heat tolerance. *miR842* and *miR846* represent another example of this type of interaction (Jia and Rock, 2013). They arise from AS of the same transcription unit. ABA was shown to reduce the functional isoform (*miR846*) and to increase the non-functional isoform (*miR842*).

Evidence of the second type of interaction emerged from transcriptomic analyses. A systemic search performed in *Arabidopsis* using annotated gene models as well as RNA sequencing data identified 354 high-confidence miRNA binding sites, and among them at least 44 (12.4%) were affected by AS (Yang et al., 2012). Vitulo et al. (2014) emphasized that such mechanism can play a role in plant response to abiotic stress, including drought. In grapevine, AS was found to affect the miRNA target site in 139 genes. In turn, miRNA coding genes have been shown to be largely affected by splicing and AS events were observed both in grapevine (Mica et al., 2010) and *Arabidopsis* (Hirsch et al., 2006), suggesting an additional level of regulation.

It is worth noting that miRNA processing and splicing events share part of their enzymatic machinery, implying a co-regulation of their regulatory pathways. For instance, it has been shown that SERRATE (SE), CAP BINDING PROTEINS (CBP) 80 and CBP20, which cooperate in the selection of alternative splice sites (Raczynska et al., 2014), also interact with DCL1 in pri-miRNA processing, retaining a dual role in both miRNA production and mRNA splicing (Laubinger et al., 2008).

Components of the ubiquitination system are also targets of some miRNAs. The most outstanding example is the PHOSPHATE2 (*PHO2*)-*miR399* circuit (Bari et al., 2006; Chiou et al., 2006) involved in Pi homeostasis. However, other studies have recently suggested that several miRNAs regulate E3 ubiquitin ligases or E2 conjugating enzymes, especially during stress-related responses (Sunkar et al., 2007; Bertolini et al., 2013; Pandey et al., 2013), here including drought stress. Interactions among small RNAs and sumoylation can also be expected in the epigenetic component of the stress tolerance. Indeed, the list of strongly sumoylated proteins upon stress include INVOLVED IN DE NOVO 2 (IDN2), a protein involved in the control of DNA modifications through small-interfering RNA-mediated maintenance methylation (Miller et al., 2013).

An intriguing interplay between ubiquitin and SUMO modifiers has been discovered in *Arabidopsis*. A subset of sumoylated proteins becomes ubiquitinated during heat stress, suggesting that bound SUMOs act as secondary degrons for directing proteins to the ubiquitin proteasome system (Miller et al., 2010). SUMO-targeted ubiquitin ligases responsible for these modifications have been found in various plant genomes, including at least three in *Arabidopsis* (Elrouby et al., 2013). Multiple signaling pathways may converge on the same target protein by multisite post-translational modifications. They may act together in complex combinatorial regulatory patterns, have antagonistic effects, or act with different timing profiles to ensure an accurate spatio-temporal regulation of the abundance and activity of the target. ABI5, a key TF in ABA signaling, offers a very intriguing example of linked post-translational modifications (Liu and Stone, 2014). At least four ubiquitin ligases mediate the degradation of ABI5 by the 26S proteasome, thus negatively regulating ABA signaling. They are the already mentioned KEG (Stone et al., 2006), DWD HYPERSENSITIVE TO ABA (DWA) 1 and DWA2 (Lee et al., 2010), and ABA-HYPERSENSITIVE DCAF1 (ABD1; Seo et al., 2014). In the absence of ABA, KEG-mediated degradation of ABI5 inhibits ABA signaling. Conversely, ABA treatment promotes KEG auto-ubiquitination and subsequent degradation, resulting in ABI5 accumulation into the nucleus and its activation via phosphorylation. The activation through phosphorylation of ABI5 is mediated by the CALCINEURIN B-LIKE INTERACTING PROTEIN KINASE (CIPK) 26. Interestingly, KEG is also able to interact with CIPK26 and to target it for 26S proteasome-mediated degradation providing a further level of control of ABI5 activity (Lyzenga et al., 2013). DWA1, DWA2, and ABD1, instead, seem implicated in the attenuation of ABA signaling, binding AB15 directly and affecting its stability in the nucleus, upon ABA treatment. To complicate this picture, sumoylation also intervenes to regulate ABI5 abundance. Indeed, ABI5 sumoylation protects ABI5 from degradation and preserves an inactive pool of ABI5 in the absence of ABA (Miura et al., 2009).

## GENETIC DIVERSITY IN POST-TRANSCRIPTIONAL AND POST-TRANSLATIONAL MODIFICATIONS

Due to the fundamental function of the described mechanisms in the plant tolerance to stress, a natural genetic diversity is expected among genotypes with different behavior in terms of adaptation to stressful conditions. However, to date few studies have been published about the exploration of allelic variations associated with post-transcriptional and post-translational mechanisms and about their phenotypic impact. Most of them concern miRNAs and ubiquitin ligases.

For AS, diversity could involve specific alterations of the splicing sites or general modifications of the splicing machinery. Some studies indicate that genotypic variability exists for AS in plants (Gan et al., 2011; Mastrangelo et al., 2012). In particular, Vitulo et al. (2014) found, in grapevine, a higher variability for AS among genotypes than among tissues and stress treatments. Moreover, they observed that the panel of SR splicing factors showed a few, but very marked differences among genotypes.

miRNA gene sequences as well as their *cis*- and *trans*-regulatory elements may carry sequence variation and polymorphism, even though they are subjected to strong purifying selection (Ehrenreich and Purugganan, 2008; Guo et al., 2008), with few examples of positive selection. Sequence variation could occur at the precursor level (pre-miRNA), thus modifying the processing efficiency and the stability of the secondary structures essential to the mature miRNA excision, in the promoter or regulatory regions, influencing the expression level of the relative miRNAs, and at the mature miRNA sequence, influencing miRNA:target interactions. The latter is a rare event under strong selective constraints since it would disrupt the regulatory network, implying a co-evolution of the miRNA and its target. Polymorphisms at miRNA target sites have also been identified and they could have the effect of destabilizing the interaction between the miRNA and the mRNA, which could consequently avoid cleavage and lead to phenotypical variations (Colaiacovo et al., 2010).

Some recent studies have emphasized miRNA polymorphisms as main players of plant domestication and adaptability to the environment. For instance, it has been shown that domestication of rice has increased the level of sequence polymorphisms in some miRNAs, highlighting the positive role of small RNA-driven regulation in crop improvement (Wang et al., 2010). Moreover, many domestication genes are TFs (Doebley et al., 2006) that are also the most abundant class of miRNA target genes, thus reinforcing the hypothesis that miRNAs play a role in domestication.

In *Arabidopsis*, SNPs have been found in many miRNA genes, both in the miRNA:miRNA* region and in the precursor region, causing phenotypic variability (Todesco et al., 2012). Sequence variation in the pre-miRNA has been shown to drive the evolution of *MIR* genes in different ecotypes of *Arabidopsis* influencing their genetic adaptation to different climatic conditions, since the mutation modifies the stability of the secondary structure that strongly depends on the temperature at which the ecotype grows (de Meaux et al., 2008). *miR164* represents a case of polymorphism affecting the miRNA:miRNA* duplex that results in different leaf shapes (Todesco et al., 2012).

Differential expression of miRNAs has also been reported. *A. thaliana* and the closely related *A. arenosa* show a dramatic difference in *miR163* accumulation due to differences in their promoter regions, in the level of hystone modifications, and in the pri-miRNA sequence, with demonstrated effects on primary transcript processing rate (Ng et al., 2011). Similar evidence, even if not explored at the molecular level, has been provided by many reports focused on the differential expression of miRNAs in different genotypes subjected to abiotic stresses (Kong et al., 2010; Barrera-Figueroa et al., 2011; Juszczak and Baier, 2012; Yin et al., 2012; Zhao et al., 2012; Li et al., 2014).

Polymorphisms in the coding sequence of ubiquitin ligases or their protein targets may cause loss on function/interaction mutations, thus causing strong phenotypic variations, as those observed for many already characterized mutants. Weak phenotypic effects are instead expected mainly due to genetic variability at the level of gene expression, due to polymorphisms in the regulatory regions of genes. Thus, examination of existing data on transcriptional/transcriptomic changes upon stress treatments, ubiquitin ligases are often found in the list of differentially expressed genes among genotypes with contrasting levels of stress tolerance (De Leonardis et al., 2007; Aprile et al., 2013). Genetic evidence indicates diversity for ubiquitination too. A F-box ubiquitin ligase has been found among the selected genes during the domestication process in maize (Doebley et al., 2006). Cloning of some QTLs has revealed ubiquitin ligases as genetic determinants of relevant traits for yield and for evolutionary fitness as well, like grain size and seed setting. Thus, the ubiquitin E3 ligase *GW2* underpins one of the major rice QTL for grain width and weight (Song et al., 2007). GW2 functions as a negative regulator of grain width and weight, indeed loss of function mutants show wider and heavier rice grains. Interestingly, *GW2* had been cloned based on a polymorphism in the promoter region severely affecting gene expression. Also for *GW5*, the second major rice QTL controlling grain width and weight, the candidate gene is involved in the ubiquitin–proteasome pathway (Weng et al., 2008). A more recent example is offered by the E3 ubiquitin ligase POLLEN TUBE BLOCKED 1 (PTB1), a positive regulator of rice panicle seed setting rate (Li et al., 2013), for which natural variation in the gene expression correlated with the trait.

Sumoylation actually is a basal cellular process, thus limited natural variability is expected. However, evidence of genetic diversity exists. A phylogenetic comparison of the SUMO conjugation system among several dicots and monocots has identified a core set of indispensable components, and provided evidence for species-specific features of the sumoylation pathway, such as copy number variation of homologous genes, and sequence polymorphisms that suggested monocot-specific variants (Novatchkova et al., 2012). Generally, SUMO ligases and proteases showed the most pronounced differences. In addition, genetic studies indicate some level of diversity for sumoylation. Indeed, SIZ1 has been associated with two major loci regulating seed yield under low phosphate conditions in oilseed rape (Shi et al., 2013).

## NEW TOOLS AND CANDIDATE GENES FOR NOVEL RESISTANCE GENOTYPES

By illustrating several examples, we have shown the extensive impact of alterations in the post-transcriptional and post-translational control systems, as well as in their targets, on tolerance to abiotic stresses in plants. Therefore, these systems provide new candidate targets to be exploited for the development of novel genotypes with improved performance when exposed to environmental adversities. Both genetic manipulation, based on heterologous gene expression, and natural/induced diversity could be exploited for this aim. Loss of function mutations or over-expression of specific components of post-transcriptional or post-translational mechanisms may generate genotypes with increased tolerance (Mazzucotelli et al., 2008). Even if improved tolerance of these engineered plants is sometimes associated with penalties in plant growth and/or yield, thus limiting their effective exploitation, some examples are very promising. For instance, the constitutive over-expression of the rice SUMO E3 ligase *OsSIZ1* in creeping bentgrass (*Agrostis stolonifera* L.) enhanced performance when plants were subjected to water deficiency and heat stress. The enhanced performance is associated with an increased overall plant growth and photosynthesis rate, more robust root growth, higher water retention and cell membrane integrity (Li et al., 2012). Plants with production of artificial miRNAs (amiRNA) have also been of some success. Indeed, improved drought tolerance was obtained in potato after amiRNA-based silencing of *CBP80*, whose corresponding protein is involved in RNA processing. High tolerance to water stress was correlated to ABA-hypersensitive stomatal closure, increased leaf stomata and trichome density. At the molecular level, *CBP80* silencing resulted in a cascade of effects, including the down-regulation of *miR159* and the subsequent up-regulation of the ABA-related TF genes *MYB33* and *MYB101* (Pieczynski et al., 2013).

Exploiting natural/induced diversity could even be of greater success. Indeed, if it were shown that post-transcriptional and post-translational modifications integrate other signaling pathways besides the main stress signal, faint variations of specific components of such modifications could modulate the impact of specific signaling pathways on the amount/activity of targets. In the future, genome re-sequencing programs will provide sequence information to mine relevant natural alleles in genes encoding the components of post-transcriptional and post-translational modifications and in their targets. Alternatively, new genome editing technologies (TALENs, Crispr/Cas9 etc.) could allow the creation of new alleles for relevant genes, followed by the identification of the most promising ones (Gaj et al., 2013). New alleles and allele patterns could then be used in molecular breeding of new, improved plant genotypes.

To support these strategies, knowledge about the molecular basis of stress-related traits must still advance through the identification of new protagonists of these post-transcriptional and post-translational modifications, new targets of their activities, and the spatio-temporal connections among different regulatory levels, as well as the natural variation existing for all of them. A deeper understanding of these components will allow the finest and most favorable tuning of mechanisms that plants have developed to regulate stress response processes.

### Conflict of Interest Statement

The authors declare that the research was conducted in the absence of any commercial or financial relationships that could be construed as a potential conflict of interest.
